# Low-dose aspirin for the prevention of preterm birth in nulliparous women: systematic review and meta-analysis

**DOI:** 10.1186/s12884-024-06413-2

**Published:** 2024-04-11

**Authors:** Xin Yan, Wei Zheng, Jia Wang, Xianxian Yuan, Guanghui Li

**Affiliations:** Division of Endocrinology and Metabolism, Department of Obstetrics, Beijing Obstetrics and Gynecology Hospital, Capital Medical University, Beijing Maternal and Child Health Care Hospital, No.251, Yaojiayuan Road, Chaoyang District, Beijing, 100026 China

**Keywords:** Aspirin, meta-analysis, Preterm birth, Systematic review

## Abstract

**Objective:**

The objective was to assess the efficacy and safety of low-dose aspirin for the prevention of preterm birth in nulliparous women.

**Data sources:**

We searched PubMed, Embase and the Cochrane Central Register of Controlled Trials (CENTRAL) from inception to June 2022.

**Study eligibility criteria:**

Randomized controlled trials that compared aspirin to placebo in nulliparous women were eligible.

**Methods:**

This study was reported in accordance with the PRISMA 2020 checklist. The primary outcomes of this study were the rates of preterm birth at less than 37 weeks and less than 34 weeks of gestation. The secondary outcomes included postpartum hemorrhage, placental abruption, cesarean section, any hypertensive disorder of pregnancy and small for gestational age. Relative risks with their 95% confidence intervals were calculated for analysis. Heterogeneity was assessed by Cochran’s Q test and Higgins’s I^2^. A random-effects model was used when I^2^ was > 50% to generate the RR and 95% CI; otherwise, a fixed-effects model was used. The risk of publication bias was assessed by funnel plots. We performed sensitivity analysis by sequentially omitting each included study to confirm the robustness of the analysis.

**Results:**

Seven studies with a total of 29,029 participants were included in this review. Six studies were assessed as having a low risk of bias or an unclear risk of bias, and one study was judged as having a high risk of bias. In nulliparous women, low-dose aspirin was associated with a significant reduction in the rate of preterm birth at less than 34 weeks of gestational age (RR 0.84,95% CI: 0.71–0.99; I^2^ = 0%; *P* = 0.04), but we did not observe a significant difference in the rate of preterm birth at less than 37 weeks of gestation (RR 0.96,95% CI: 0.90–1.02; I^2^ = 31%; *P* = 0.18). Low-dose aspirin was associated with a significant increase in the rates of postpartum hemorrhage (RR 1.32,95% CI: 1.14–1.54; I^2^ = 0%; *P* = 0.0003), placental abruption (RR 2.18,95% CI: 1.10–4.32; I^2^ = 16%; *P* = 0.02) and cesarean section (RR 1.053, 95% CI: 1.001–1.108; I^2^ = 0%; *P* = 0.05) in nulliparous women. We also did not observe a significant effect of low-dose aspirin on the rates of any hypertensive disorder of pregnancy (RR 1.05, 95% CI: 0.96–1.14; I^2^ = 9%; *P* = 0.28) or small for gestational age (RR 0.96, 95% CI: 0.91–1.02; I^2^ = 0%; *P* = 0.16) in nulliparous women. Funnel plots indicated that no significant publication bias existed in this meta-analysis. Except for preterm birth at less than 34 weeks of gestation, placental abruption and cesarean section, the sensitivity analysis showed similar results, which confirmed the robustness of this meta-analysis.

**Conclusions:**

Low-dose aspirin might reduce the risk of preterm birth at less than 34 weeks of gestation in nulliparous women. The use of low-dose aspirin in nulliparous women increased the risk of postpartum hemorrhage and might increase the risk of placental abruption and cesarean section.

**Supplementary Information:**

The online version contains supplementary material available at 10.1186/s12884-024-06413-2.

## Introduction

Preterm birth (PTB), associated with infant mortality and morbidity, is an important public health problem [1]. In 2015, PTB was the leading cause of under-5 and neonatal deaths in 194 WHO member states (1.055 million deaths and 0.944 million deaths, respectively) [2]. Due to the immaturity of several organ systems, surviving preterm infants are at increased risk for cerebral palsy, blindness, deafness, intraventricular hemorrhage, necrotizing enterocolitis, respiratory distress syndrome, jaundice, and intellectual disability [3, 4]. Additionally, preterm infants are more likely to develop adult-onset chronic diseases such as obesity, diabetes, and hypertension. The costs to the health care system for caring for preterm infants and their long-term effects are enormous. Moreover, PTB can cause psychological and physical distress and illness for the mother and the community [5]. There are a wide variety of treatments to prevent PTB, including cervical cerclage and progesterone [6–9]. However, two-thirds of patients with PTB have no apparent risk factors to explain its occurrence, which means that prevention of PTB may not be effective and it remains important to identify new preventive strategies for PTB [10]. 

Aspirin belongs to the family of nonsteroidal anti-inflammatory drugs (NSAIDs) and acts as an inhibitor of two cyclooxygenase isoenzymes (COX-1 and COX-2), thereby reducing the synthesis of prostaglandins and thromboxane [11]. Aspirin was first reported to prevent the recurrence of preeclampsia in 1978, and its effectiveness in preventing preeclampsia has been confirmed by multiple randomized trials and meta-analyses [12]. Currently, some health care organizations recommend that women with risk factors for preeclampsia use low-dose aspirin (LDA) to prevent or delay the onset of preeclampsia [13–17]. Because PTB partly resembles the pathophysiology of uteroplacental ischemic lesions in preeclampsia, LDA may also be used to prevent PTB [18, 19]. 

Evidence from randomized trials suggested that LDA was effective in preventing preeclampsia and associated PTB in women at high risk for preeclampsia [20, 21]. In 2017, a meta-analysis also reported that antiplatelet drugs reduced the rate of spontaneous PTB in pregnant women at risk of preeclampsia [22]. However, whether LDA can be used to prevent PTB in women not at risk of preeclampsia is unclear. On a population basis, nulliparity was strongly associated with PTB [10], which raised concerns about whether LDA can be used to prevent PTB in nulliparous women. In 2018 and 2020, a secondary analysis of a randomized trial of low-dose aspirin for the prevention of preeclampsia in nulliparous women and the ASPIRIN trial showed that LDA reduced the incidence of PTB in nulliparous women [23, 24], which might support the idea that LDA could be used to prevent PTB in nulliparous women.

The objective of this systematic review and meta-analysis was to assess the efficacy and safety of LDA for the prevention of PTB in nulliparous women.

## Materials and methods

This was a systematic review and meta-analysis of randomized controlled trails that evaluated the impact of LDA on PTB in nulliparous women. The protocol was registered in PROSPERO (CRD42022343951).

### Data sources and search strategy

PubMed, EMBASE and the Cochrane Central Register of Controlled Trials (CENTRAL) were searched from inception to June 2022 using MeSH terms and keywords related to aspirin and preterm birth in pregnant women (Shown in supplementary Table S1). The references of other systematic reviews and meta-analyses were evaluated to identify potentially relevant references. No language restriction was applied.

### Eligibility criteria

Studies were included if they (1) were randomized controlled trials; (2) included one group of nulliparous women who received LDA at any dose that commenced at any time during pregnancy and another group that received placebo; and (3) reported data on the prevalence of preterm birth. Studies were excluded if they (1) included pregnant women who had the following issues: allergy or contraindication to aspirin, chronic kidney disease, diabetes, infections, and medical conditions for which LDA therapy is currently indicated (e.g., hypertension, systemic lupus erythematosus or antiphospholipid syndrome); (2) were review articles, editorials, case reports, conference abstracts, or non-placebo-controlled studies; and (3) were secondary analyses that used the same population data as other original studies.

### Study selection and data extraction

All studies were independently selected by two reviewers [XY (Xin Yan) and JW] by initially screening abstracts and titles. Then, the full text of all potentially eligible studies was retrieved and read in detail by two reviewers [XY (Xin Yan) and JW] to assess inclusion eligibility. Relevant data were extracted for each study by two reviewers [XY (Xin Yan) and JW] independently, including the first author’s name, publication year, sample size, inclusion criteria and exclusion criteria, intervention, onset and duration, compliance, and outcomes. Microsoft Excel was used for data extraction. Any disagreements during study selection and data extraction were resolved by discussion among all authors [XY (Xin Yan), JW, XY (Xianxian Yuan), WZ, GL]. WZ and GL checked for accuracy.

### Outcome measures

The primary outcomes of this study were the rates of preterm birth at less than 37 weeks and less than 34 weeks of gestation. Secondary outcomes included postpartum hemorrhage (> 500 ml) [25], placental abruption, cesarean section, any hypertensive disorder of pregnancy and small for gestational age (birthweight below the 10th centile by gestational age and sex) [26]. 

### Quality evaluation

Preferred reporting items for systematic reviews and meta-analysis (PRISMA) tool were used to assess the quality of this review (Shown in supplementary Table S2) [27]. The risks of bias in each included study were independently assessed according to the Cochrane Handbook criteria by two reviewers [XY (Xin Yan) and WZ] [28]. 

### Data synthesis and statistical analyses

RevMan 5.4 and STATA 16.0 were used for statistical analysis. For dichotomous outcomes, relative risks (RRs) with their 95% confidence intervals (CIs) were calculated for analysis [29]. Heterogeneity was assessed by Cochran’s Q test and Higgins’s I^2^ [30, 31]. A random-effects model was used when I^2^ was > 50% to generate the RR and 95% CI; otherwise, a fixed-effects model was used. We summarized the data using forest plots. The risk of publication bias was assessed by funnel plots. We performed sensitivity analysis by sequentially omitting each study to ensure the robustness of the analysis.

## Results

### Study selection

**Fig. 1:**
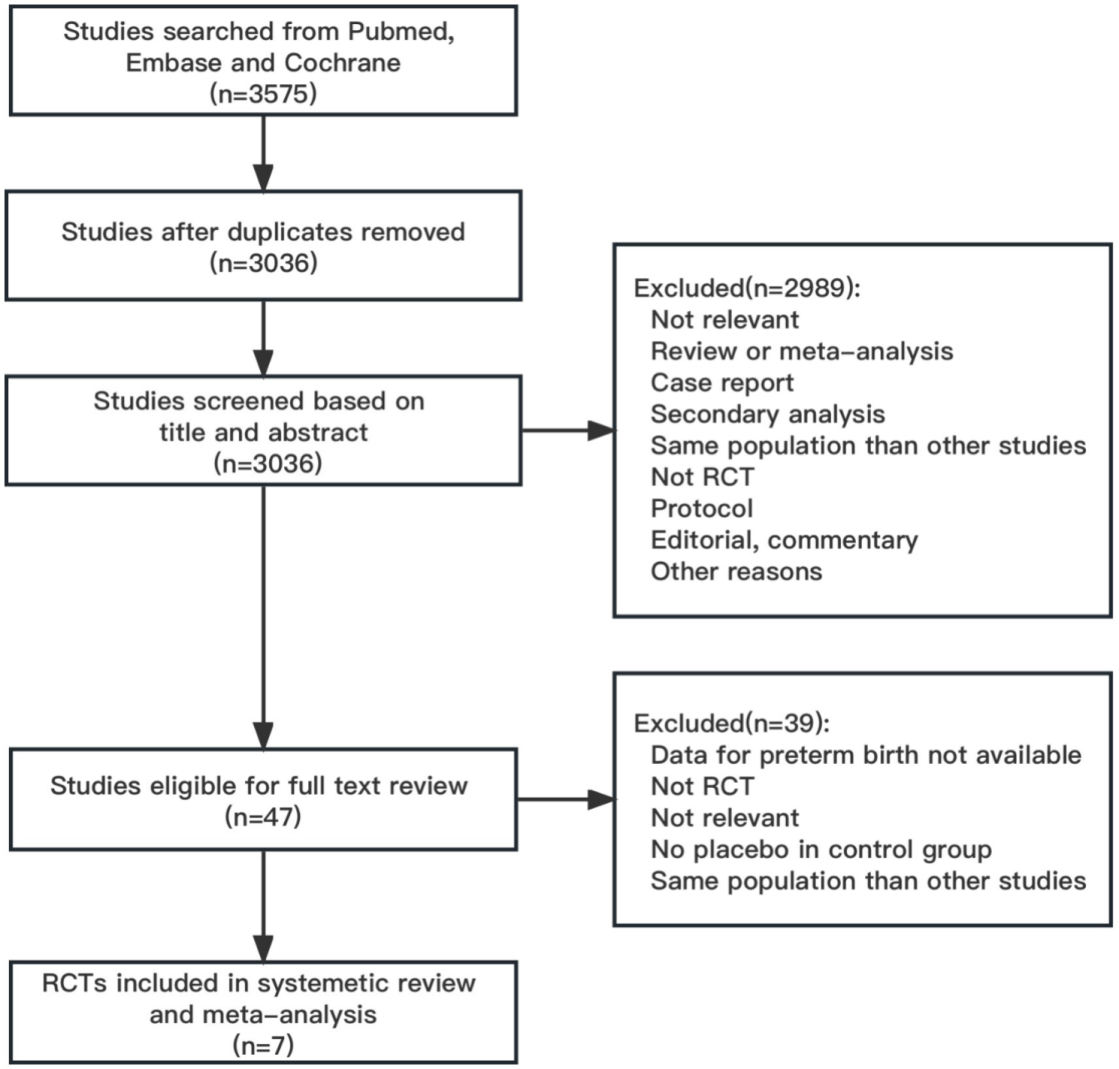
Flowchart of study selection

We retrieved 3575 citations from PubMed, Embase and the Cochrane Central Register of Controlled Trials up to June 2022. A total of 3568 citations were excluded, including duplicate publications, reviews and meta-analyses, irrelevant studies, case reports, secondary analyses, studies including the same population as another study, protocols, non-RCTs, editorials, commentaries, and studies for which PTB data were not available. We excluded 3 additional studies in which there was no placebo in the control group [32–34]. Finally, 7 studies with a total of 29,029 participants were included in this review [24, 35–40]. A flowchart of the study selection process is shown in Fig. 1.

### Study characteristics

The characteristics of the included studies are shown in Table 1. Six out of the seven studies reported the effect of LDA on the rates of PTB at less than 37 weeks of gestation, and three out of the seven studies reported the effect of LDA on the rates of PTB at less than 34 weeks of gestation. The data for the rates of PTB at less than 37 weeks of gestation included the data for the rates of PTB at less than 34 weeks of gestation. There were two studies both reported the rates of PTB at less than 37 weeks of gestation and the rates of PTB at less than 34 weeks of gestation and these data are presented separately in each study [24, 36]. Regarding postpartum hemorrhage, placental abruption and cesarean section, four, three and six out of the seven studies reported the effect of aspirin, respectively. For any hypertensive disorder of pregnancy and small for gestational age, all studies and five out of the seven studies reported the effect of aspirin, respectively.

**Table 1:** Characteristics of included studies

Study	Participants	N	Compliance	Interventions		
				Aspirin	Control	Onset and duration
Sibai 1993	We enrolled nulliparous women seeking prenatal care who were 13 to 25 weeks pregnant if their blood pressure was below 135/85 mmHg and they had no proteinuria on testing with a dipstick (sensitivity, less than 30 mg per deciliter if the dipstick result is zero or trace). Women with chronic hypertension, renal disease, diabetes mellitus, and other medical illnesses were excluded.	3135	Compliance with treatment was assessed by asking about tablet intake and by counting tablets. The estimated percentage of the prescribed tablets taken averaged 83% in the aspirin group and 84% in the placebo group. 72% of the women in the aspirin group and 74% of the women in the placebo group took 80% or more of the tablets prescribed between the start of the study and delivery.	60 mg daily	Placebo	From recruitment until the onset of labor
Hauth 1993	Nulliparous, healthy, and with a singleton gestation at between 20 and 22 weeks’ gestation.	604	We assessed patient compliance by a capsule count at each 2-week visit. The mean compliance index, with a maximum of 100, was 94.5 (SD ± 5.4) in the aspirin group and 94.4 (SD ± 7.53) in the placebo group (*p* = 0.43, not significant).	60 mg daily	Placebo	From 24 weeks until delivery.
Davies 1995	Healthy, nulliparous women with a high hemoglobin concentration in the second trimester.	118	At each antenatal visit women were asked about the compliance. They described that compliance was excellent but without specific data.	75 mg daily	Placebo	From 18 weeks until delivery.
Golding 1998	Nulliparous women enrolled between 12 and 32 weeks of gestation.	6275	Compliance was measured by counting the remaining tablets and was recorded only if bottles were available at each visit and remaining pills were consistent with expectation. The authors considered that compliance in this study was poor.	60 mg daily	Placebo	From recruitment until delivery
Rotchell 1998	All women attending antenatal clinics between 12 and 32 weeks of gestation were eligible, if without specific contraindications to aspirin and unlikely to deliver immediately.	3647	Compliance was measured by calendar sheets. Each treatment pack contained seven one-month calendar sheets. 55% of women reported taking tablets for more than 80% of the time.	75 mg daily	Placebo	From recruitment until delivery
Subtil 2003	Women were eligible if they were nulliparous (no previous delivery at or after 22 weeks), at a gestational age between 14 and 20.6 weeks, planned to continue prenatal care and give birth in the participating facility and provided written informed consent.	3274	Two methods were used to assess the compliance: counting remaining packets and salicyluric acid assays during the seventh month of pregnancy in some patients. The last day of treatment was known for 88.7% of the patients. Of them, 83.7% reported treatment compliance of at least 80%.	100 mg daily	Placebo	From 14–20 weeks untill 34 weeks.
Hoffman 2020	Nulliparous women between 14 and 40 years of age with an ultrasound confirming gestational age and singleton viable pregnancy in multi-country. Women were excluded if had any of the following by medical history: allergy or contraindication to aspirin, previously taken aspirin therapy for more than 7 days during this pregnancy, multiple gestations, history of more than two first trimester losses, or medical conditions for which low-dose aspirin therapy is currently indicated (e.g. diabetes and hypertension).	11,976	A pre-packaged 2-week medication allotment was exchanged every 2 weeks by study personnel to assess compliance. Overall adherence to medication or placebo defined as taking ≥ 90% of the prescribed medications was high (MITT population overall 84.9%: aspirin 85.3% vs. placebo 84.4%).	81 mg daily	Placebo	Initiated between 6 weeks and 0 days and 13 weeks and 6 days until 36 weeks and 0 days of pregnancy

### Risk of bias of included studies

**Fig. 2:**
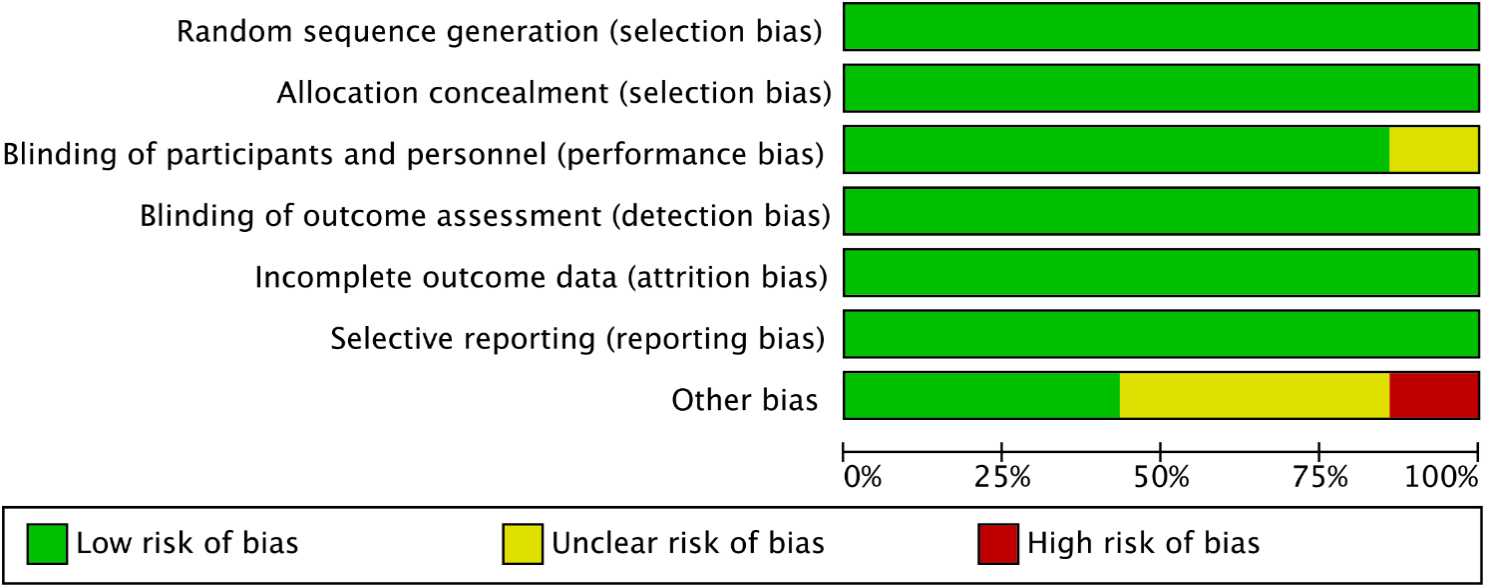
Risk of bias for included studies

**Fig. 3:**
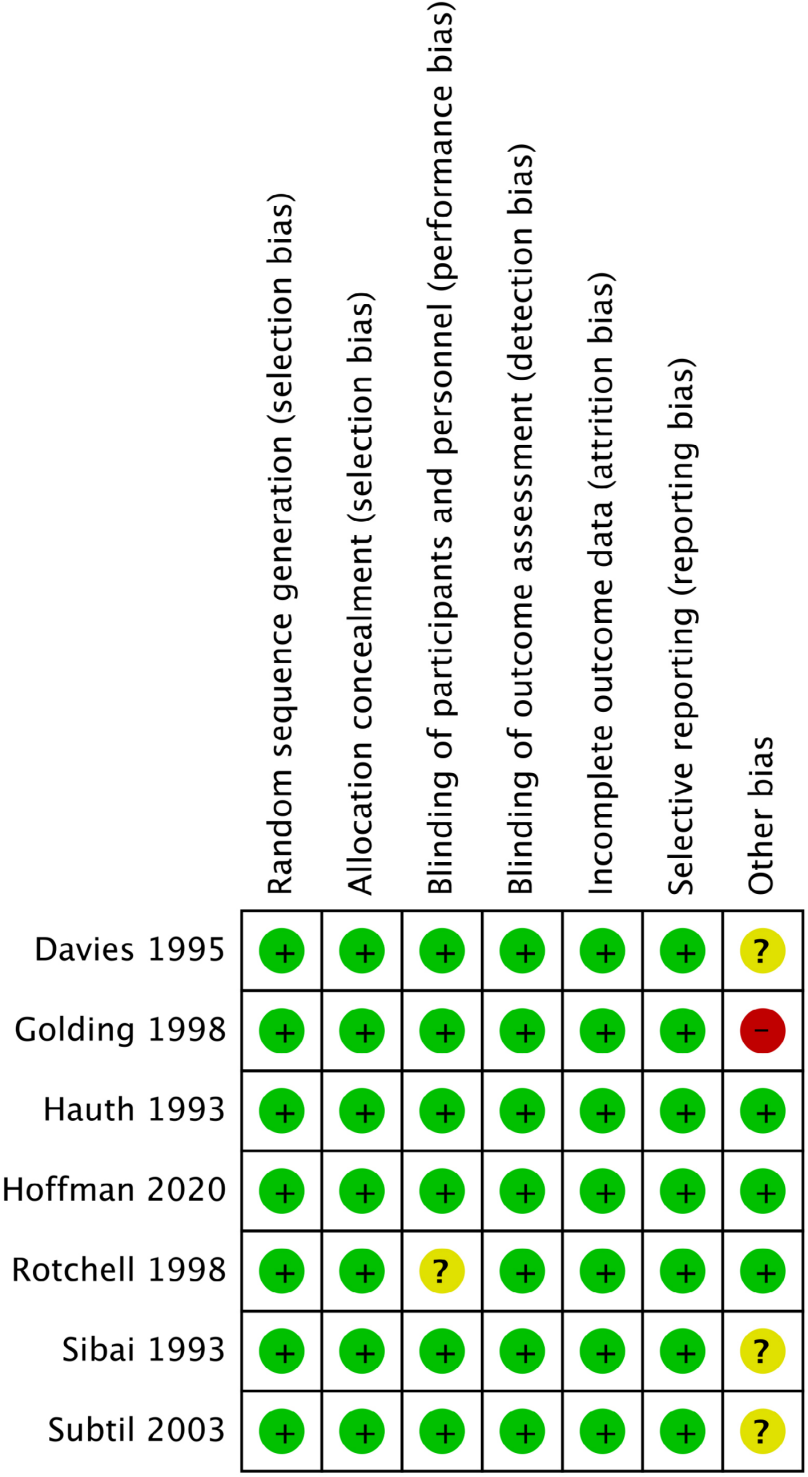
Risk of bias summary for included studies

The quality assessment of the included studies according to the Cochrane Handbook criteria is shown in Fig.2 and Fig.3; 6 studies were assessed as having a low risk of bias or an unclear risk of bias, and 1 study was judged as having a high risk of bias.

**Fig. 4:**
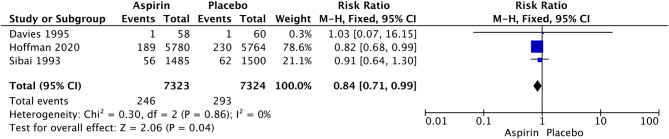
Forest plot of the effect of aspirin on PTB less than 34 weeks of gestation

### Synthesis of results

The synthesis of results for each of the assessed outcomes in the included studies is shown in Table 2.

**Table 2:** Summary statistics using the fixed-effects model in assessing the effect of aspirin on pregnancy outcomes in nulliparous women

Outcomes	Trials, n	Participants, n	Fixed effect, relative risk (95% CI)	P value	I^2^, %
Preterm birth less than 37 weeks of gestation	6	26,086	0.96 (0.90, 1.02)	0.18	31
Preterm birth less than 34weeks of gestation	3	14,647	0.84 (0.71, 0.99)	0.04	0
Postpartum hemorrhage	4	24,143	1.32 (1.14, 1.54)	0.0003	0
Placental abruption	3	6354	2.18 (1.10, 4.32)	0.02	16
Cesarean section	6	24,588	1.05 (1.00, 1.11)	0.05	0
Any hypertensive disorder of pregnancy	7	26,168	1.05 (0.96, 1.14)	0.28	9
Small for gestational age	5	18,005	0.96 (0.91, 1.02)	0.16	0

CI: confidence interval

**Fig. 5:**
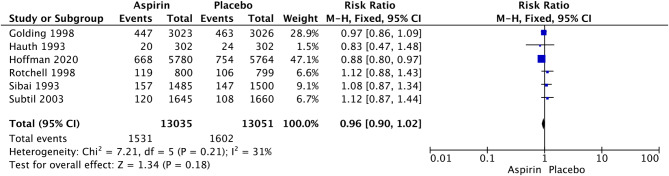
Forest plot of the effect of aspirin on PTB less than 37 weeks of gestation

In nulliparous women, LDA was associated with a significant reduction in the rate of PTB at less than 34 weeks of gestational age (RR 0.84, 95% CI: 0.71–0.99; I^2^ = 0%; *P* = 0.04; Fig. 4), but we did not observe a significant difference in the rate of PTB at less than 37 weeks of gestation (RR 0.96, 95% CI: 0.90–1.02; I^2^ = 31%; *P* = 0.18; Fig. 5). Given that the definition of PTB before 37 weeks of gestation encompasses PTB before 34 weeks of gestation, this study further examined the relationship between LDA and the incidence of PTB between 34 and 37 weeks of gestation. The analysis revealed that there was no statistically significant difference in the effect of LDA on the incidence of PTB between 34 and 37 weeks of gestation (RR 1.01, 95% CI: 0.78–1.32; I^2^ = 68%; *P* = 0.91; Supplementary Figure S1).

**Fig. 6:**
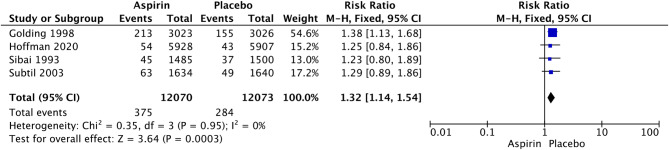
Forest plot of the effect of aspirin on postpartum hemorrhage

**Fig. 7:**
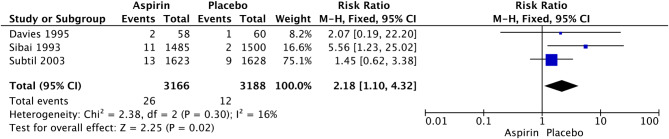
Forest plot of the effect of aspirin on placental abruption

**Fig. 8:**
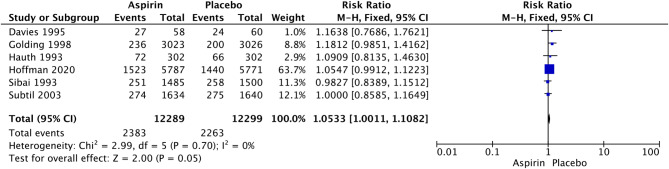
Forest plot of the effect of aspirin on cesarean section

**Fig. 9:**
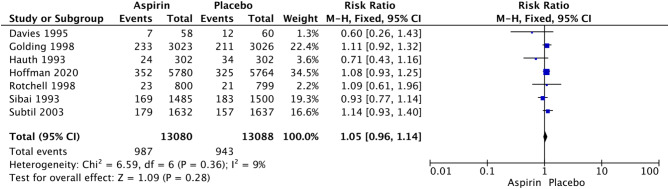
Forest plot of the effect of aspirin on any hypertensive disorder of pregnancy

**Fig. 10:**
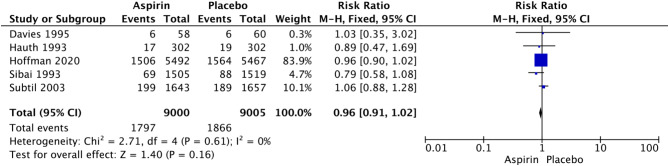
Forest plot of the effect of aspirin on small for gestational age

LDA was associated with a significant increase in the rates of postpartum hemorrhage (RR 1.32, 95% CI: 1.14–1.54; I^2^ = 0%; *P* = 0.0003; Fig. 6), placental abruption (RR 2.18, 95% CI: 1.10–4.32; I^2^ = 16%; *P* = 0.02; Fig. 7) and cesarean section (RR 1.053, 95% CI: 1.001–1.108; I^2^ = 0%; *P* = 0.05; Fig. 8) in nulliparous women. We did not observe a significant effect of LDA on the rates of any hypertensive disorder of pregnancy (RR 1.05, 95% CI: 0.96–1.14; I^2^ = 9%; *P* = 0.28; Fig. 9) or small for gestational age (RR 0.96, 95% CI: 0.91–1.02; I^2^ = 0%; *P* = 0.16; Fig. 10) in nulliparous women.

### Sensitivity analyses

Sensitivity analysis was performed by omitting studies sequentially (Table 3). For preterm birth at less than 37 weeks of gestation, the pooled RR ranged from 0.94 (95% CI: 0.88, 1.01) to 1.02 (95% CI: 0.93, 1.12). For preterm birth at less than 34 weeks of gestation, the pooled RR ranged from 0.82 (95% CI: 0.68, 0.99) to 0.91 (95% CI: 0.64, 1.30), and when the study by Hoffman was omitted, the estimated effect became nonsignificant (RR 0.91, 95% CI: 0.64, 1.30). For postpartum hemorrhage, the pooled RR ranged from 1.26 (95% CI: 1.00, 1.58) to 1.34 (95% CI: 1.13, 1.57). For placental abruption, the pooled RR ranged from 1.51 (95% CI: 0.68, 3.35) to 4.40 (95% CI: 1.26, 15.41), and when the study by Sibai et al. was omitted, the estimated effect became nonsignificant (RR 1.51, 95% CI: 0.68, 3.35). The pooled RR for cesarean section ranged from 1.04 (95% CI: 0.99, 1.10) to 1.06 (95% CI: 1.01–1.12), which indicated heterogeneity, and when the study by Golding or by Hoffman et al. was omitted, the estimated effect became nonsignificant (RR 1.04, 95% CI: 0.99, 1.10 and RR 1.05, 95% CI: 0.96, 1.15; independently). For any hypertensive disorder of pregnancy, the pooled RR ranged from 1.03 (95% CI: 0.93, 1.15) to 1.08 (95% CI: 0.98, 1.18). For small for gestational age, the pooled RR ranged from 0.95 (95% CI: 0.90, 1.01) to 0.97 (95% CI: 0.92–1.03).

**Table 3:** Sensitivity analyses by sequential omission of each included study

Study omitted	RR (95% CI) for preterm birth less than 37 weeks of gestation	RR (95% CI) for preterm birth less than 34 weeks of gestation	RR (95% CI) for postpartum hemorrhage	RR (95% CI) for placental abruption	RR (95% CI) for cesarean section	RR (95% CI) for any hypertensive disorder of pregnancy	RR (95% CI) for small for gestational age
Davies 1995	/	0.84 (0.71, 0.99)	/	2.19 (1.08, 4.47)	1.05 (1.00, 1.11)	1.05 (0.97, 1.15)	0.96 (0.91, 1.02)
Golding 1998	0.95 (0.88, 1.02)	/	1.26 (1.00, 1.58)	/	1.04 (0.99, 1.10)	1.03 (0.94, 1.14)	/
Hauth 1993	0.96 (0.90, 1.02)	/	/	/	1.05 (1.00, 1.11)	1.06 (0.97, 1.16)	0.96 (0.91, 1.02)
Hoffman 2020	1.02 (0.93, 1.12)	0.91 (0.64, 1.30)	1.34 (1.13, 1.57)	/	1.05 (0.96, 1.15)	1.03 (0.93, 1.15)	0.97 (0.83, 1.13)
Rotchell 1998	0.94 (0.88, 1.01)	/	/	/	/	1.05 (0.96, 1.14)	/
Sibai 1993	0.94 (0.88, 1.01)	0.82 (0.68, 0.99)	1.34 (1.14, 1.57)	1.51 (0.68, 3.35)	1.06 (1.01, 1.12)	1.08 (0.98, 1.18)	0.97 (0.92, 1.03)
Subtil 2003	0.94 (0.88, 1.01)	/	1.32 (1.13, 1.57)	4.40 (1.26, 15.41)	1.06 (1.01, 1.12)	1.03 (0.94, 1.13)	0.95 (0.90, 1.01)
All studies	0.96 (0.90, 1.02)	0.84 (0.71, 0.99)	1.32 (1.14, 1.54)	2.18 (1.10, 4.32)	1.05 (1.00, 1.11)	1.05 (0.96, 1.14)	0.96 (0.91, 1.02)

CI: confidence interval; RR: relative risk

### Publication bias

Funnel plots for each outcome in this review were created to qualitatively evaluate publication bias (Supplementary Figures S2-8). All funnel plots seem to be symmetric, which indicates that no significant publication bias existed in this meta-analysis.

### Comment

#### Principal findings

This meta-analysis showed that low-dose aspirin might reduce the risk of PTB at less than 34 weeks of gestation in nulliparous women. However, its impact on reducing the overall risk of PTB at less than 37 weeks of gestation is not statistically significant. Further analysis reveals that there is no statistically significant difference in the effect of LDA on the incidence of PTB between 34 and 37 weeks of gestation. The use of LDA in nulliparous women increased the risk of postpartum hemorrhage and might increase the risk of placental abruption and cesarean section. We also did not observe a significant effect of LDA on the incidence of any hypertensive disorder of pregnancy or small for gestational age in nulliparous women.

PTB is a syndrome with multiple etiological risk factors and phenotypic characteristics [41]. Research on preventive interventions for PTB would be meaningless and inexplicable without a deep understanding of this. Generally, PTB can be divided into early PTB and late PTB according to gestational age. PTB at less than 34 weeks of gestation may have different pathophysiological mechanisms compared to those between 34 and 37 weeks, potentially leading to differential effects of LDA. Research has indicated that early PTB is more likely to be associated with inflammation [18, 42]. In this meta-analysis, the ability of low-dose aspirin to prevent PTB at less than 34 weeks of gestation might be due to its anti-inflammatory properties. In 2018, Andrikopoulou et al. reported similar results that low-dose aspirin reduced the risk of spontaneous PTB at less than 34 weeks of gestation but not 37 weeks of gestation in healthy nulliparous women [23]. In addition, it is important to emphasize that only considering gestational age to classify preterm births also has limitations. The clinical phenotype, such as one or more features of maternal, fetal, placental, and delivery manifestations, should also be considered [43]. However, all studies included in our meta-analysis simply classified early and late PTB only by gestational age, which might lead to heterogeneity among studies. This might also partly explain the unstable results of preterm birth at less than 34 weeks of gestation when performing sensitivity analysis in our meta-analysis. Furthermore, it was observed that the results of the meta-analysis examining the effect of LDA on the incidence of PTB between 34 and 37 weeks of gestation demonstrated a high level of heterogeneity. This heterogeneity may be attributable to variations in the study populations, the timing of the studies conducted, and the dosages of aspirin administered. Therefore, to elucidate the impact of LDA on the incidence of PTB within this specific gestational age, additional research is warranted for a more comprehensive understanding.

LDA is recommended for women with risk factors for preeclampsia to prevent or delay the onset of preeclampsia. In recent years, several cost-effectiveness analyses have suggested that the routine use of aspirin to prevent preeclampsia in all women was associated with lower costs and greater health benefits compared with the use of aspirin only in women with risk factors after screening [44–46]. A cost-effectiveness analysis indicated that routine aspirin use to prevent preeclampsia in low-risk nulliparous women was also a preferred strategy [47]. In contrast, our meta-analysis showed that low-dose aspirin was ineffective in preventing preeclampsia in nulliparous women. The mechanism behind the effect of aspirin on preeclampsia in low-risk nulliparous women is unclear. Hence, the universal use of low-dose aspirin to prevent preeclampsia in women without risk factors is not acceptable [48]. Abuse of low-dose aspirin in nulliparous women might pose additional risks. Further research is needed to address these issues.

In our meta-analysis, the use of LDA in nulliparous women increased the risk of postpartum hemorrhage and potentially increased the risk of placental abruption and cesarean section. In contrast, a meta-analysis revealed that there was no increased risk of postpartum hemorrhage in nulliparous women who took low-dose aspirin, which might be due to the lack of several studies in this meta-analysis [49]. A recent population-based cohort study from Sweden also showed that aspirin use during pregnancy was associated with an increased risk for postpartum hemorrhage [50]. Andrikopoulou et al. found that placental abruption was more common in nulliparous women receiving low-dose aspirin than in those receiving placebo (OR 10.0; 95% CI: 1.16–100.0) [23]. A secondary analysis of the effects of aspirin in gestation and reproduction trials by Eubanks et al. showed that preconception-initiated LDA was not associated with the risk of cesarean section (RR 1.02; 95% CI: 0.98–1.07) compared with placebo [51]. Overall, the safety of aspirin use in healthy nulliparous women has not been proven, and further studies are needed.

#### Comparison with the existing literature

In 2017, van Vliet et al. conducted a meta-analysis suggesting that antiplatelet drugs reduced the rate of spontaneous PTB in pregnant women at risk of preeclampsia [22]. However, with further subgroup analysis, this meta-analysis revealed that aspirin was not effective in preventing spontaneous preterm birth at less than 37 and 34 weeks of gestation in nulliparous women who were at risk of preeclampsia. In addition, this meta-analysis did not demonstrate the effectiveness of aspirin in preventing PTB in nulliparous women who were not at risk of preeclampsia.

Man et al. performed a meta-analysis in healthy nulliparous women with singleton pregnancies and found that taking aspirin reduced the relative risk of preterm birth less than 34 weeks of gestation by 50% but there were no significant differences in preterm birth less than 37 weeks of gestation, which was similar to our study [49]. It is worth noting that the meta-analysis by Man et al. and our study differ in terms of search strategies and primary outcomes. As a result, two studies included in our meta-analysis were not captured in their analysis [39, 40]. Moreover, the meta-analysis conducted by Man et al. included two secondary analyses that shared the same population with an original study. Interestingly, they excluded the original study from their analysis. This approach differs from our study, where we included the original study and excluded the secondary analyses to prevent duplication of data [23, 36, 52]. Furthermore, it should be noted that the meta-analysis by Man et al. included three studies that had no treatment in the control group. However, we made a different decision in our meta-analysis and chose to exclude these studies from our analysis due to concerns related to the study design. The three studies had small sample sizes, and we believed that the absence of a placebo in the control group may have introduced performance and detection bias. Therefore, by excluding these studies, we aimed to minimize potential bias and ensure the robustness of our findings. Additionally, it is important to mention that the meta-analysis conducted by Man et al. did not report on the effects of aspirin on outcomes such as placental abruption and cesarean section. In contrast, our meta-analysis specifically examined these outcomes and included relevant studies to provide a comprehensive analysis of the effects of aspirin. By including these outcomes, we aimed to assess the broader impact of aspirin on maternal and fetal health.

### Limitations of the study

To our knowledge, this is the first meta-analysis in which the primary outcome was the effect of aspirin on the prevention of PTB in nulliparous women. The most obvious limitation of this meta-analysis was that not all included studies reported the incidence of PTB, and only 3 studies reported the incidence of PTB at less than 34 weeks of gestation [24, 36, 37]. Due to the small number of studies included in this meta-analysis, the effect of onset timing and the dose of LDA on the rate of PTB was not evaluated. Furthermore, the study did not differentiate between spontaneous and iatrogenic preterm births due to a lack of adequate studies. Sensitivity analysis demonstrated that the results of this meta-analysis were robust, except for PTB at less than 34 weeks of gestation, placental abruption and cesarean section. Two of the seven studies did not distinguish between singleton and multiple pregnancies, which caused unclear bias for this meta-analysis, but considering the very small number of women with multiple pregnancies, we decided to include these two studies in our meta-analysis [36, 40]. Finally, most studies (6/7) had a low or an unclear risk of bias, with one study having a high risk of bias due to poor compliance [38]. 

## Conclusions and implications

The results of this meta-analysis suggested that prophylactic LDA might reduce the risk of PTB at less than 34 weeks of gestation in nulliparous women. LDA might be beneficial for specific phenotypes of PTB, but given the phenotypic heterogeneity of PTB, how to identify women who would benefit from prophylactic LDA remains to be elucidated. Future work will require large prospective randomized controlled trials to evaluate the preventive effect of LDA on specific PTB phenotypes. Moreover, we found that LDA increased the risk of postpartum hemorrhage and might increase the risk of placental abruption and cesarean section in nulliparous women, which implied that prophylactic use of LDA to prevent PTB in all nulliparous women must be done with great caution.

Given the wide range of enrollment periods during pregnancy, extra caution is warranted when interpreting the conclusions of this meta-analysis. Currently, there is a scarcity of studies on the administration of aspirin for the prevention of preterm birth in nulliparous women. Further research is needed to examine the potential benefits of aspirin in this population. Specifically, future studies should investigate the optimal timing, dosage, and discontinuation of aspirin, as well as its effectiveness in preventing various subtypes of preterm birth.

In summary, although the meta-analysis indicated that prophylactic use of LDA might prevent preterm birth at less than 34 weeks of gestation in nulliparous women, much work remains to be done to determine the impact of LDA on PTB [53]. Potential risks and benefits need to be considered when using low-dose aspirin to prevent PTB in nulliparous women.

### Electronic supplementary material

Below is the link to the electronic supplementary material.

Supplementary Material 1

Supplementary Material 2

Supplementary Material 3

Supplementary Material 4

Supplementary Material 5

Supplementary Material 6

Supplementary Material 7

Supplementary Material 8

Supplementary Material 9

Supplementary Material 10

## Data Availability

All data generated or analysed during this study are included in this published article and are available from the corresponding author on reasonable request.

## Abbreviations

PTB: preterm birth
LDA: low dose aspirin
RR: relative risks
CI: confidence intervals
