# Supplementary material for: Low-dose aspirin for the prevention of preterm birth in nulliparous women: systematic review and meta-analysis
Source: BMC Pregnancy Childbirth. 2024 Apr 11;24:260. doi: 10.1186/s12884-024-06413-2 (PMC11008052; doi:10.1186/s12884-024-06413-2)
Supplement: Supplementary file 1 — Supplementary Material 1 [file 12884_2024_6413_MOESM1_ESM.docx]

Supplementary Table S1 Search strategy

| Database | Search terms | Results |
| --- | --- | --- |
| Pubmed | ((("Aspirin"[Mesh]) OR (((((((((Acetylsalicylic Acid) OR (Acid, Acetylsalicylic)) OR (2-(Acetyloxy)benzoic Acid)) OR (Acylpyrin)) OR (Ecotrin)) OR (Micristin)) OR (Polopiryna)) OR (Solprin)) OR (Zorprin))) AND ((((((((((((((Preterm Birth) OR (Birth, Premature)) OR (Births, Premature)) OR (Premature Births)) OR (Birth, Preterm)) OR (Births, Preterm)) OR (Preterm Births)) OR (Labor, Premature Obstetric)) OR (Preterm Labor)) OR (Labor, Preterm)) OR (Premature Obstetric Labor)) OR (Labor, Premature)) OR (Premature Labor)) OR ((((((((Primiparity) OR (Nulliparity)) OR (Pregnant Woman)) OR (Woman, Pregnant)) OR (Women, Pregnant)) OR (Nulliparous Women)) OR (Pregnancy)) OR (pregnant)))) AND ((clinical[Title/Abstract] AND trial[Title/Abstract]) OR clinical trials as topic[MeSH Terms] OR clinical trial[Publication Type] OR random*[Title/Abstract] OR random allocation[MeSH Terms] OR therapeutic use[MeSH Subheading]) | 2710 |
| Embase | ('premature labor'/exp OR 'preterm birth' OR 'preterm labor' OR 'pregnant woman'/exp OR 'pregnancy'/exp) AND ('acetylsalicylic acid'/exp OR aspirin OR '2-(acetyloxy)benzoic acid' OR acylpyrin OR ecotrin OR micristin OR polopiryna OR solprin OR zorprin) AND ('randomized controlled trial'/exp) | 361 |
| Cochrane Library | ([mh "Aspirin"] OR (Acetylsalicylic Acid):ti,ab,kw OR (Acylpyrin):ti,ab,kw OR (Ecotrin):ti,ab,kw OR (Micristin):ti,ab,kw OR (Polopiryna):ti,ab,kw OR (Solprin):ti,ab,kw OR (Zorprin):ti,ab,kw) AND ([mh "Premature Birth"] OR ("preterm birth"):ti,ab,kw OR ("preterm labor"):ti,ab,kw OR [mh "Pregnant Women"] OR [mh "Pregnancy"] OR (pregnan*):ti,ab,kw) | 504 |
