# Supplementary figures and images for: Low-dose aspirin for the prevention of preterm birth in nulliparous women: systematic review and meta-analysis
Source: BMC Pregnancy Childbirth. 2024 Apr 11;24:260. doi: 10.1186/s12884-024-06413-2 (PMC11008052; doi:10.1186/s12884-024-06413-2)

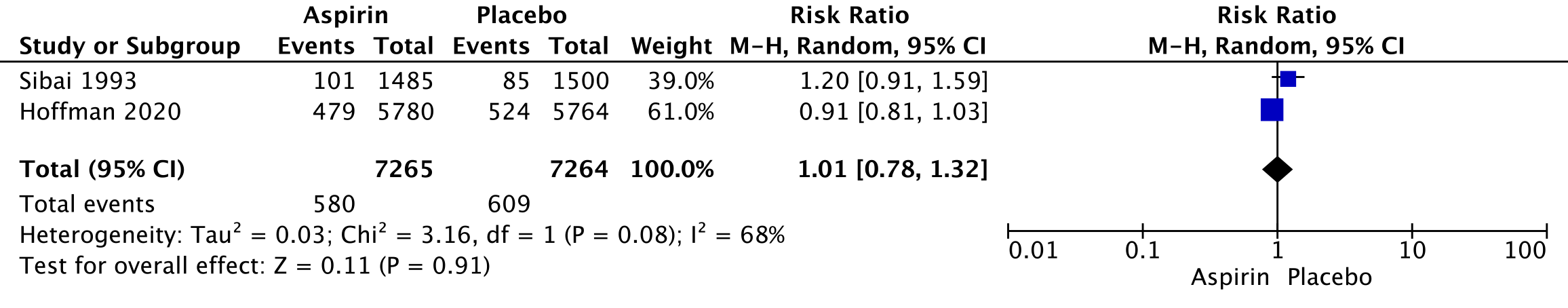

Supplement: Supplementary file 3 — Supplementary Material 3 [file 12884_2024_6413_MOESM3_ESM.tif]

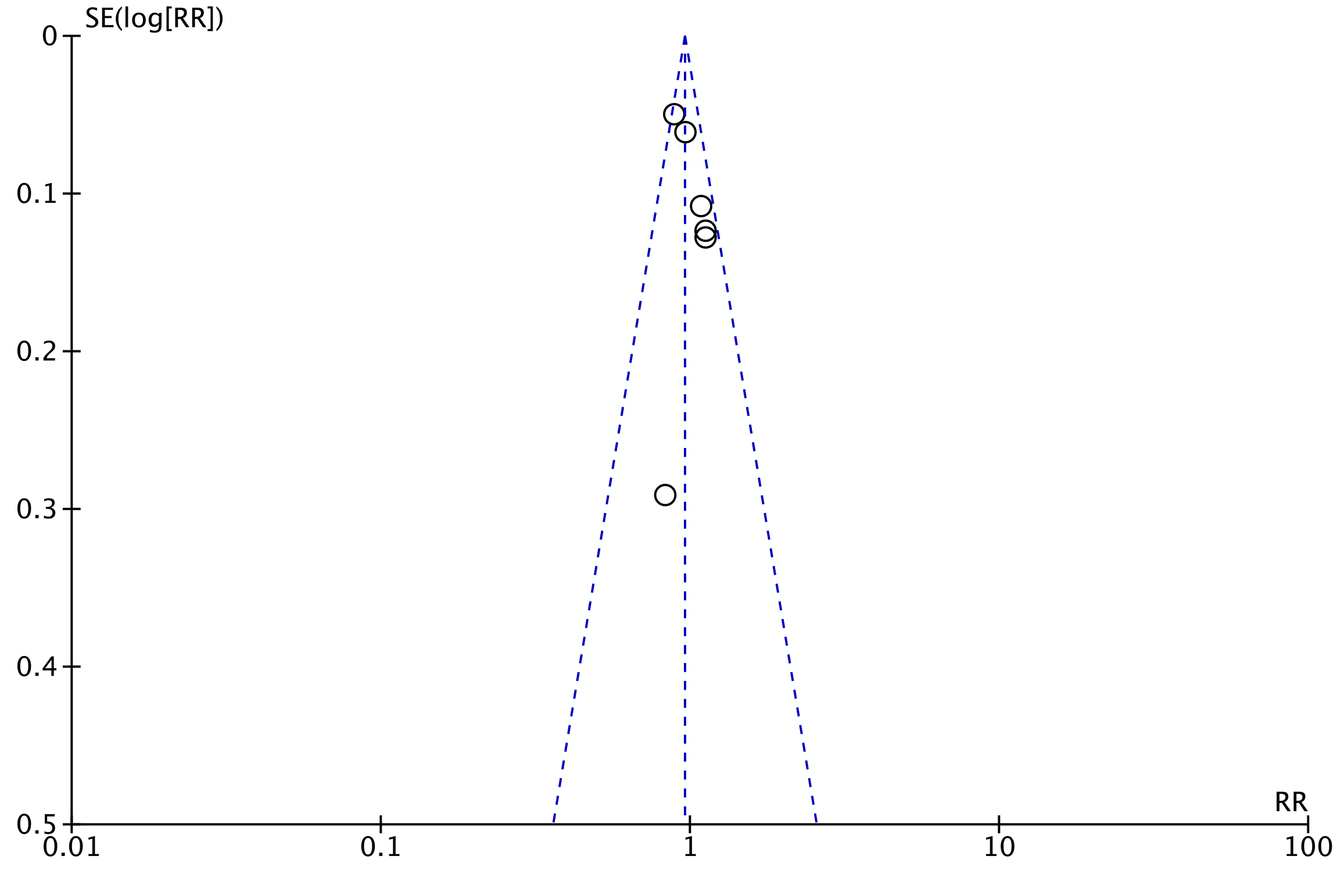

Supplement: Supplementary file 4 — Supplementary Material 4 [file 12884_2024_6413_MOESM4_ESM.tif]

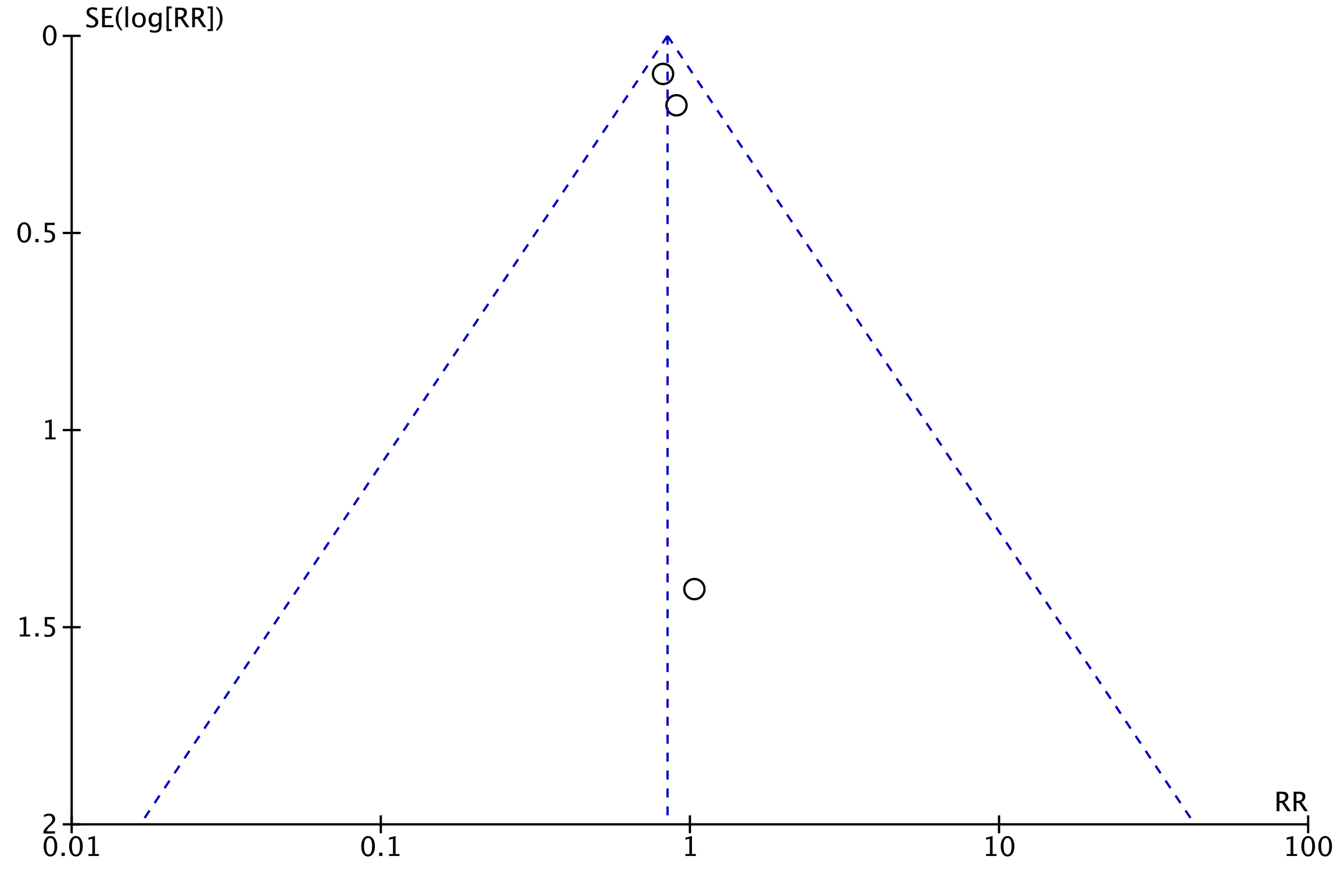

Supplement: Supplementary file 5 — Supplementary Material 5 [file 12884_2024_6413_MOESM5_ESM.tif]

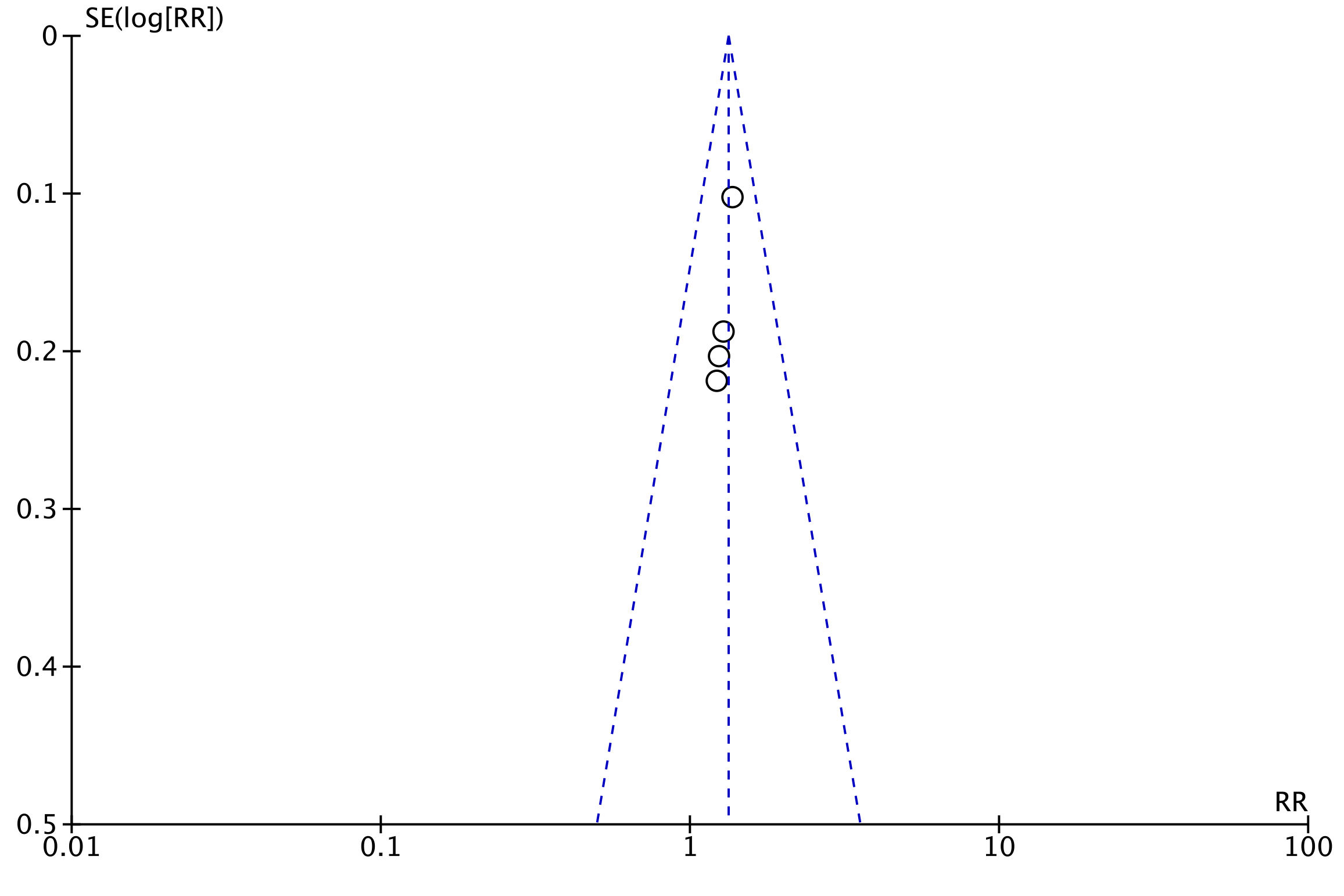

Supplement: Supplementary file 6 — Supplementary Material 6 [file 12884_2024_6413_MOESM6_ESM.tif]

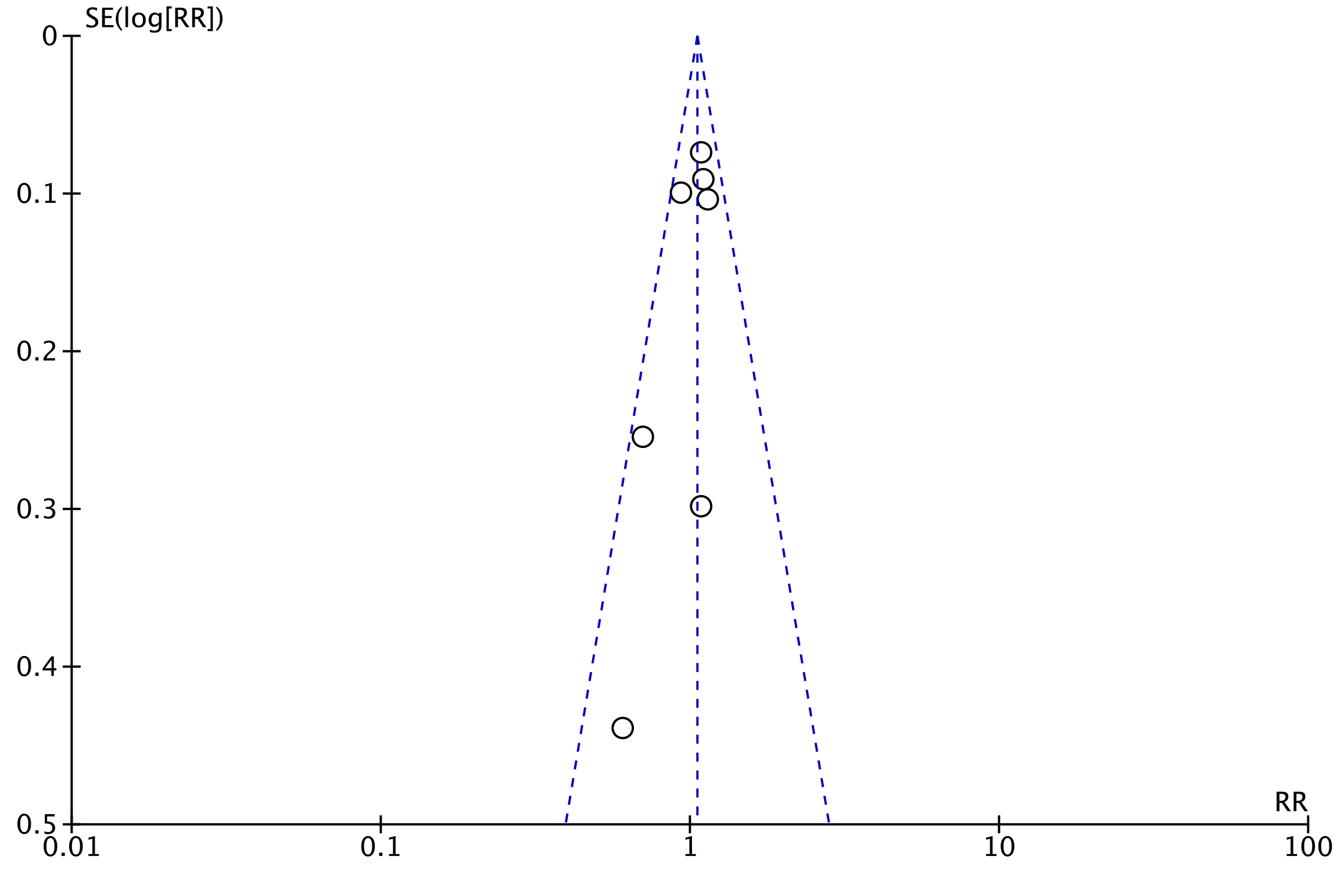

Supplement: Supplementary file 7 — Supplementary Material 7 [file 12884_2024_6413_MOESM7_ESM.tif]

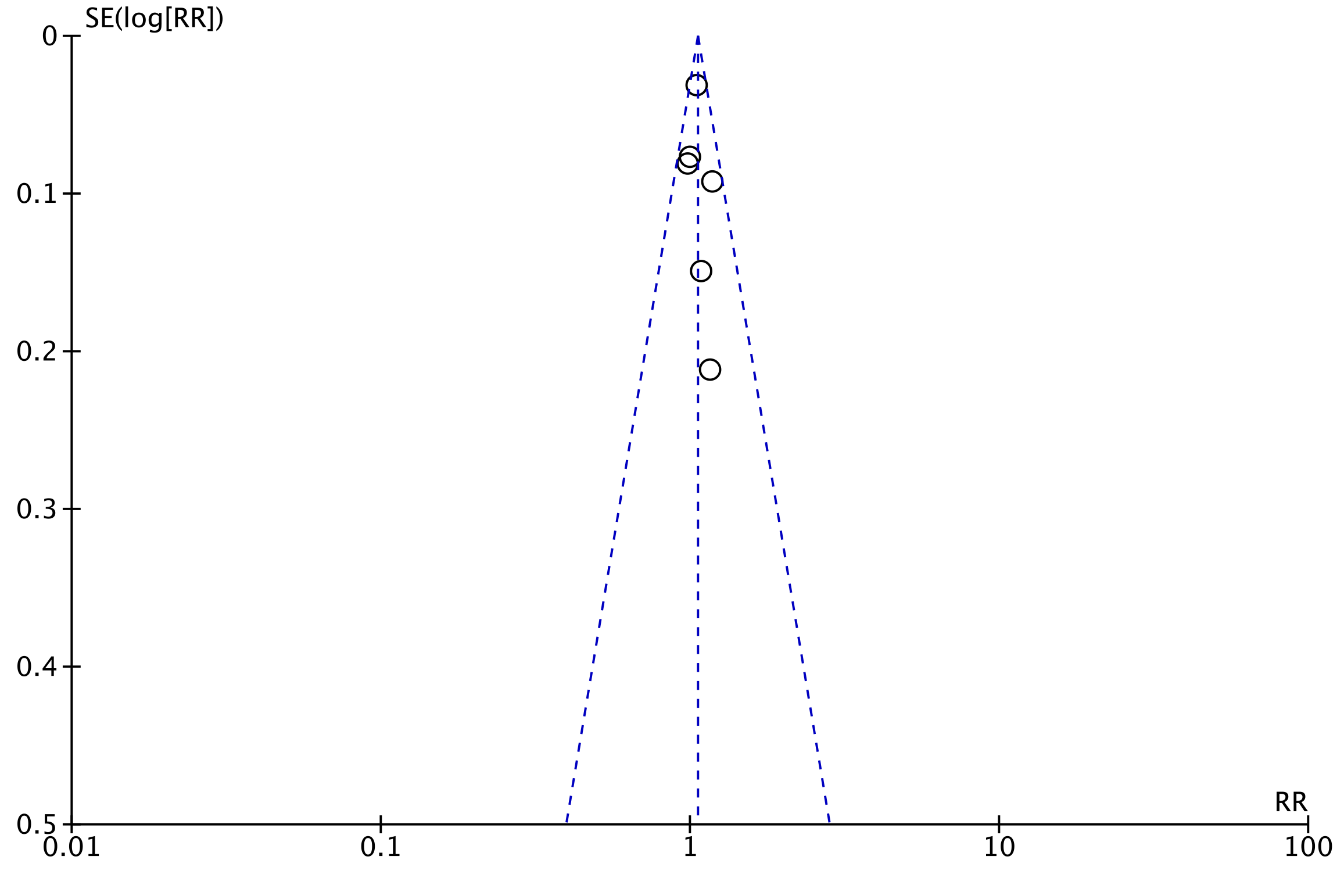

Supplement: Supplementary file 8 — Supplementary Material 8 [file 12884_2024_6413_MOESM8_ESM.tif]

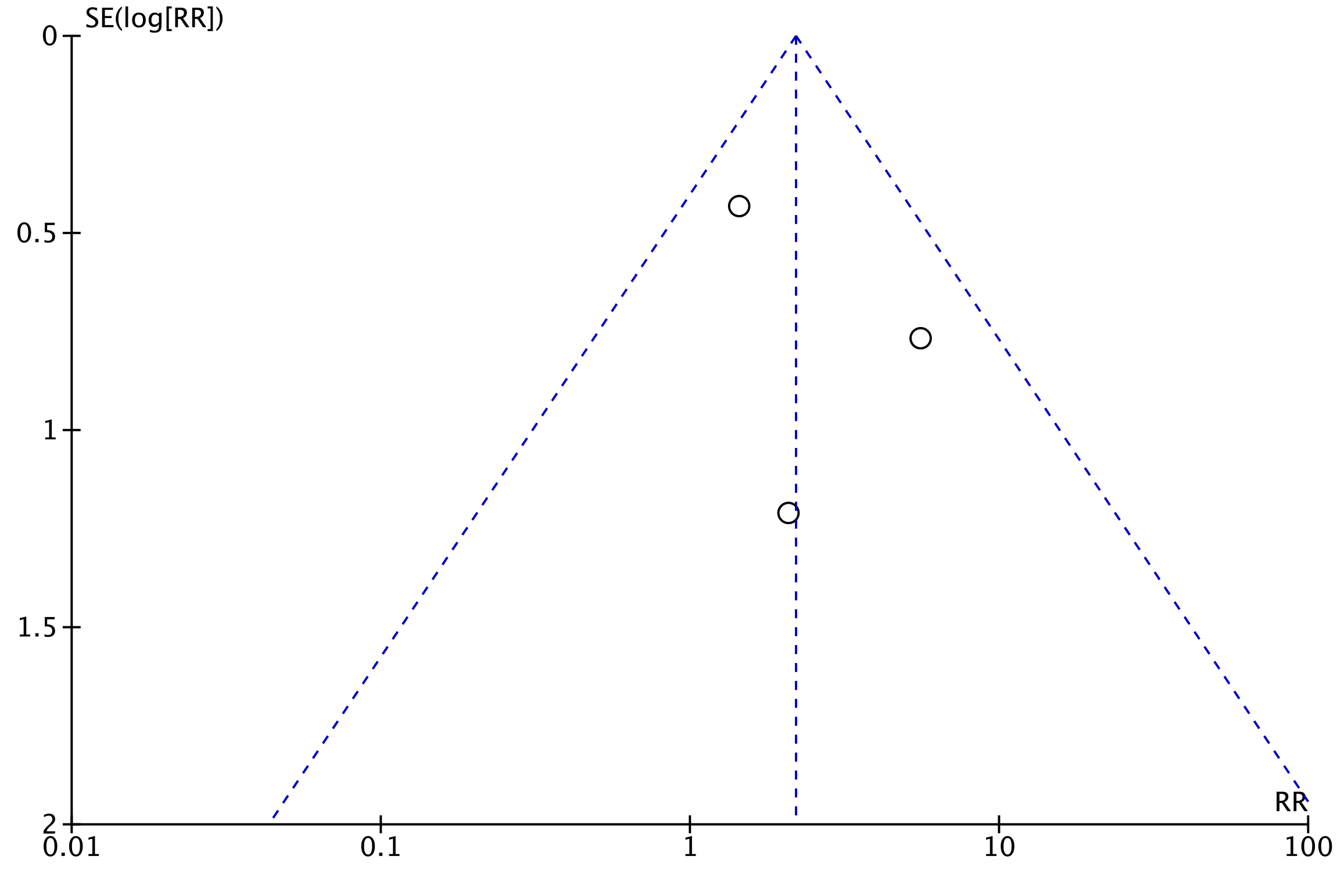

Supplement: Supplementary file 9 — Supplementary Material 9 [file 12884_2024_6413_MOESM9_ESM.tif]

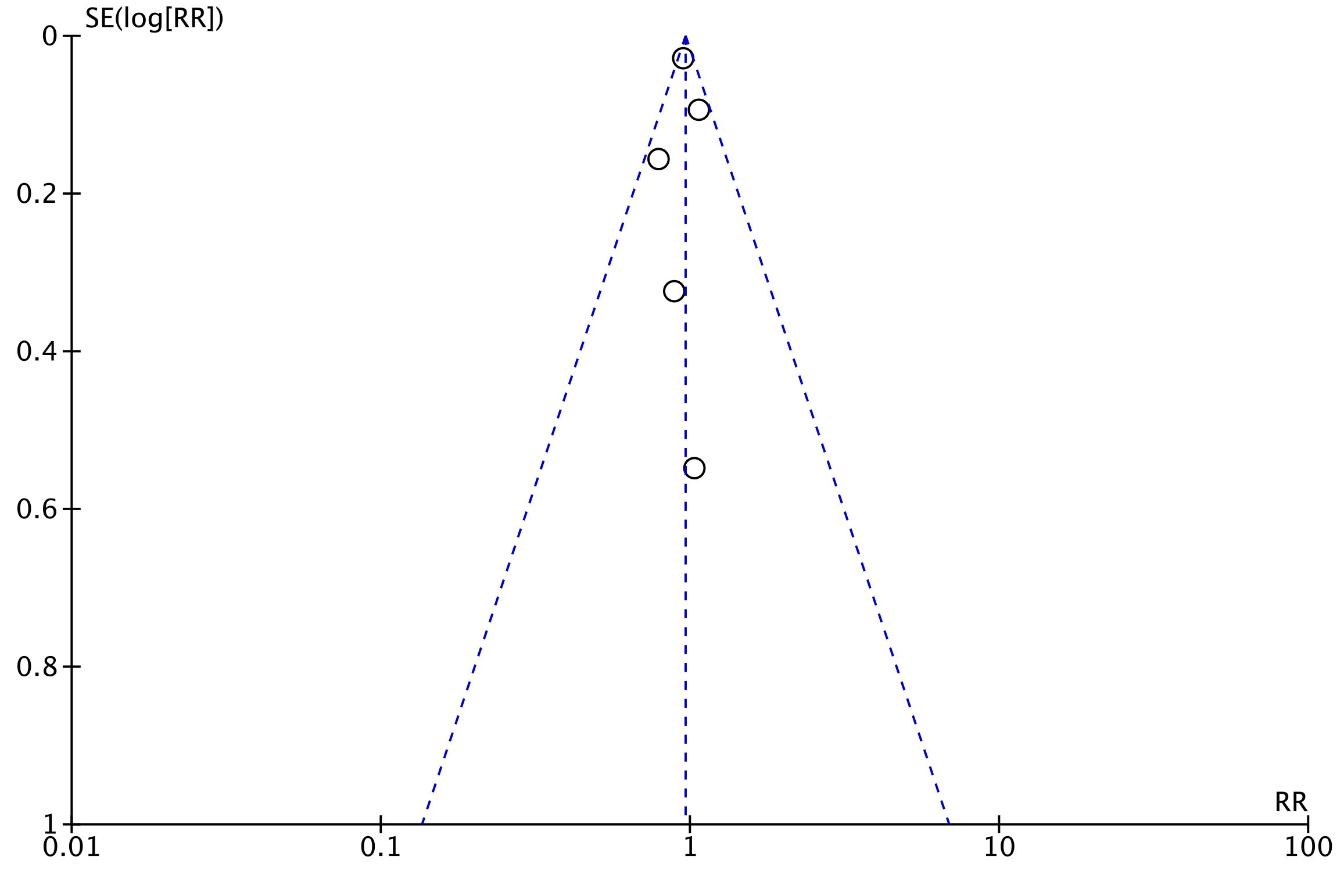

Supplement: Supplementary file 10 — Supplementary Material 10 [file 12884_2024_6413_MOESM10_ESM.tif]
